# Growth Performance, Cytokine Expression, and Immune Responses of Broiler Chickens Fed a Dietary Palm Oil and Sunflower Oil Blend Supplemented With L-Arginine and Varying Concentrations of Vitamin E

**DOI:** 10.3389/fvets.2020.00619

**Published:** 2020-10-15

**Authors:** Jannatara Khatun, Teck Chwen Loh, Hooi Ling Foo, Henny Akit, Kabirul I. Khan

**Affiliations:** ^1^Department of Animal Science, Faculty of Agriculture, Universiti Putra Malaysia, Serdang, Malaysia; ^2^Department of Animal Science and Nutrition, Chattogram Veterinary and Animal Sciences University, Chattogram, Bangladesh; ^3^Institute of Tropical Agriculture and Food Security, Universiti Putra Malaysia, Serdang, Malaysia; ^4^Department of Bioprocess Technology, Faculty of Biotechnology and Biomolecular Sciences, Universiti Putra Malaysia, Serdang, Malaysia; ^5^Institute of Bioscience, Universiti Putra Malaysia, Serdang, Malaysia; ^6^Department of Genetics and Animal Breeding, Chattogram Veterinary and Animal Sciences University, Chattogram, Bangladesh

**Keywords:** cytokine expression, immune function, dietary oils, vitamin E, serum immunoglobulin, broiler chickens

## Abstract

This study set out to examine the combined effects of the supplementation of a dietary palm oil (PO) and sunflower oil (SO) blend, 0. 25% L-Arginine (L-Arg), and different levels of vitamin E (Vit E) on growth performance, fat deposition, cytokine expression, and immune response in broilers. A total of 216 1-day-old male broiler chicks (Cobb500) were randomly distributed into six dietary groups as follows: Diet 1: 6% palm oil (negative control); Diet 2: PO and SO blend (4% palm oil and 2% sunflower oil) + 0.25% L-Arg (positive control); Diet 3: (PO and SO blend + 0.25% L-Arg) + 20 mg/kg Vit E; Diet 4: (PO and SO blend + 0.25% L-Arg) + 50 mg/kg Vit E; Diet 5: (PO and SO blend + 0.25% L-Arg) + 100 mg/kg Vit E; and Diet 6: (PO and SO blend + 0.25% L-Arg) + 150 mg/kg Vit E. Weight gain and serum IgG and IgM increased while feed conversion ratio, fat deposition, and plasma cholesterol decreased in broilers fed Vit E with the oil blend and L-Arg, compared to those fed the negative control (Diet 1). Expression of IFN and TNF-α were reduced, whereas TGF-ß1 was up-regulated as the level of Vit E increased in the broiler diets. In summary, the combination of oil blend, L-Arg, and Vit E at a level of 50 mg/kg increased the performance and altered the expression of cytokines that may positively influence immune function in broiler chickens.

## Introduction

Dietary unsaturated fat supplementation in broiler diets leads to higher levels of available metabolizable energy owing to its greater solubility and digestibility when compared to saturated fat; this produces more energy at a lower expense and therefore increases productivity (1, 2). Studies show that a combination of plant oils is more beneficial for performance and has a more positive effect on serum parameters and fatty acid deposition in broilers than a single oil (3). Moreover, supplementation of unsaturated fats (a blend of palm oil and sunflower oil) in the diet beneficially alters the fatty acid profile of broiler muscle. As a result, cholesterol content is decreased in the meat (4) with subsequent health benefits to consumers. A high intake of meat containing highly saturated fat and cholesterol has been linked to a higher incidence of disease, particularly cardiovascular disease (5). Besides the health benefits on meat composition, dietary unsaturated fats and/or combination oils can reduce abdominal fat and total body fat deposition in chickens and rats when compared to saturated fats (1, 6). Excessive deposition of abdominal fat in chickens leads to a loss of dietary energy, which can decrease carcass yield and negatively affect its acceptability to the health-conscious consumer (7). Thus, fat or oil containing both unsaturated fatty acids and saturated fatty acids would be a valuable option for broiler production.

Arginine (Arg) is one of the most versatile and essential amino acids in chickens and it must be provided in their diet due to a lack of renal argininosuccinate synthetase and argininosuccinate lyase. Arg is required for the regulation of protein synthesis (8), tissue repair, and growth, and it also plays a role in decreasing body fat deposition in ducks (9) and chickens (10). Therefore, chickens, particularly broilers, have a requirement for Arg and are extremely dependent on the dietary intake of this amino acid (11). Arg also plays an important role as a precursor of nitric oxide (NO), which controls lipogenesis and energy partitioning. We previously reported that a diet containing 0.25% L-Arg and an oil blend (4% palm oil and 2% sunflower oil) had a complementary effect on the suppression of lipogenic genes such as acetyl–CoA carboxylase (ACC) and fatty acid synthetase (FAS), and reduced fat deposition in broilers (12). Azimi Youvalari et al. (13) suggest that adding 0.2% Arg beyond NRC (1994) requirements in broiler diets results in energy splitting toward protein deposition and decreased abdominal fat pads. Moreover, dietary Arg improves immune response by enhancing nitric oxide (NO) release from macrophages (14, 15). Hence, nutritional modifications are recommended to decrease fat and cholesterol accumulation and improve muscle yield to meet the prevailing health requirements of consumers.

Despite the benefits, the inclusion of dietary unsaturated fats, mostly polyunsaturated fatty acids (PUFAs), can increase lipid oxidation and unfavorably influence the color and flavor of meat (16), decrease the performance of chickens (17), and may encourage oxidative challenges that can affect health (18). The harmful effects of feeding unsaturated fat can be overcome by including antioxidants in the diet. Vit E (α-tocopherol) acts as an important antioxidant and can be used in the diet as an effective means of inhibiting lipid oxidation. It acts as an immunomodulator, shields membranes from free radical attack via the production of NO, and enhances immune function (19, 20). Furthermore, Vit E supplementation has been reported to improve liver function and increase the number of white blood cells and red blood cells, thus increasing immune status in broilers (21). Furthermore, a significant interactive effect between oil blends, L-Arg, and Vit E on meat quality has recently been reported (22). In our previous study (12), convincing results were obtained in terms of performance, fat deposition, fatty acid profile, and blood lipid profile in broilers fed a combination of palm oil (PO) and sunflower oil (SO; 4:2) with L-Arg (0.25%). However, a combined effect of different levels of Vit E in the latter diet on performance, cytokine expression, and immune response has not been previously explored. Thus, a diet including the above mentioned oil combination with L-Arg (0.25%) was chosen and supplemented with different levels of Vit E to test the hypothesis that concurrent supplementation of the PO and SO blend, L-Arg, and Vit E will improve broiler performance, cytokine expression, and immune function. Therefore, the study was conducted to assess this diet regarding growth performance, carcass characteristics, blood lipid profile, serum immunoglobulin, and splenic cytokine expression in broiler chickens.

## Materials and Methods

### Birds, Experimental Diets, and Management

All procedures followed the protocols endorsed by the Institutional Animal Care and Use Committee of Research Policy at Universiti Putra Malaysia (UPM/IACUC/AUP-R081/2016). A total of 216 1-day-old male broiler (Cobb 500) chicks were purchased from a commercial hatchery in Malaysia. The birds were immediately weighed, marked, and randomly divided into six dietary groups (treatments). Each dietary treatment contained six replicates with six birds in each replication. The experimental diets were: Diet 1: 6% palm oil (negative control); Diet 2: PO and SO blend (4% palm oil and 2% sunflower oil) + 0.25% L-Arg (positive control); Diet 3: (PO and SO blend + 0.25% L-Arg) + 20 mg/kg Vit E; Diet 4: (PO and SO blend + 0.25% L-Arg) + 50 mg/kg Vit E; Diet 5: (PO and SO blend + 0.25% L-Arg) + 100 mg/kg Vit E); and Diet 6: (PO and SO blend + 0.25% L-Arg) + 150 mg/kg Vit E. Arg was added as L-Arg monohydrochloride (99.6% L-Arg; Herbstore Ltd., USA), and Vit E was added in the form of DL-α-tocopherol acetate. Diets were formulated to meet the nutrient requirements for broiler chickens (23) by using FeedLIVE software (FeedLIVE 1.52., Thailand). The broilers were reared for six weeks; they were vaccinated at 7 and 14 days against infectious bronchitis (IB) and Newcastle disease (ND), respectively, and at 21 days against infectious bursal diseases (IBD). All broilers were fed a starter diet from 1 to 21 days of age and a finisher diet from 22 to 42 days of age; these diets are presented in Tables 1, 2, respectively.

**Table 1:** Feed composition and calculated nutrient contents of the experimental diets fed to broiler chickens during the starter period.

**Ingredient (g/kg)**	Dietary treatments^*^					
	**Diet 1 (control)**	**Diet 2 (positive control)**	**Diet 3**	**Diet 4**	**Diet 5**	**Diet 6**
Corn	441.8	435.1	435.1	435.1	435.1	435.1
Soybean meal	312.4	329.7	329.7	329.7	329.7	329.7
Palm oil (PO)	60.0	40.0	40.0	40.0	40.0	40.0
Sunflower oil (SO)	0.0	20.0	20.0	20.0	20.0	20.0
Wheat bran	38.6	39.5	39.5	39.5	39.5	39.5
Wheat pollard	75.3	70.5	70.5	70.5	70.5	70.5
Fish meal	39.6	27.9	27.9	27.9	27.9	27.9
L-Lysine	2.0	2.0	2.0	2.0	2.0	2.0
DL-Methionine	2.0	2.0	2.0	2.0	2.0	2.0
DCP^1^	15.0	17.9	17.9	17.9	17.9	17.9
Calcium carbonate	5.0	4.6	4.6	4.6	4.6	4.6
Choline chloride	0.8	0.8	0.8	0.8	0.8	0.8
Salt	3.0	3.0	3.0	3.0	3.0	3.0
Mineral premix^2^	1.5	1.5	1.5	1.5	1.5	1.5
Vitamin premix^3^	1.5	1.5	1.5	1.5	1.5	1.5
Antioxidant^4^	0.2	0.2	0.2	0.2	0.2	0.2
Toxin binder^5^	1.3	1.3	1.3	1.3	1.3	1.3
Arginine	0.0	2.5	2.5	2.5	2.5	2.5
Vit E (mg/kg)	–	–	20.0	50.0	100.0	150.0
**Chemical composition(Calculated)^6^**						
ME (MJ/kg)	12.9	12.9	12.9	12.9	12.9	12.9
CP (g/kg)	210	210	210	210	210	210
Fat (g/kg)	82.5	82.0	82.0	82.0	82.0	82.0
Fiber (g/kg)	42.9	43.6	43.6	43.6	43.6	43.6
Calcium (g/kg)	10.2	10.2	10.2	10.2	10.2	10.2
Available P (g/kg)	4.60	4.60	4.60	4.60	4.60	4.60
Lysine (g/kg)	13.3	13.3	13.3	13.3	13.3	13.3
Methionine (g/kg)	5.50	5.40	5.40	5.40	5.40	5.40
Arginine (g/kg)	13.8	16.3	16.3	16.3	16.3	16.3
Meth + cyst (g/kg)	8.80	8.70	8.70	8.70	8.70	8.70
Tryptophan (g/kg)	2.60	2.60	2.60	2.60	2.60	2.60
α-tocopherol (analyzed, mg/kg)	40.7	37.8	50.7	78.4	124	165

1 *Di-calcium phosphate*.

2 *Vitamin premix contained: retinol 2 mg, α-tocopherol 0.02 mg, cholecalciferol 0.03 mg, menadione 1.33 mg, thiamin 0.83 mg, cobalamin 0.03 mg, riboflavin 2.0 mg, biotin 0.03 mg, folic acid 0.33 mg, niacin 23.30 mg, pantothenic acid 3.75 mg, and pyridoxine 1.33 mg*.

3 *Mineral premix contained: Fe 120 mg, Zn 100 mg, Mn 150 mg, Cu 20 mg, Mg 12 mg, Co 0.6 mg, Se 0.20 mg*.

4 *Antioxidant contained: butylatedhydroxyanisole (BHA)*.

5 *Toxin binder contained: natural hydrated sodium calcium aluminum silicates (HSCAS)*.

6 *The rations were formulated using FeedLIVE software (Thailand)*.

* *Diet 1: 6% palm oil (negative control); Diet 2: PO and SO blend (4% palm oil and 2% sunflower oil) + 0.25% L-Arg (positive control); Diet 3: (PO and SO blend + 0.25% L-Arg + 20 mg/kg Vit E); Diet 4: (PO and SO blend + 0.25% L-Arg+ 50 mg/kg Vit E); Diet 5: (PO and SO blend + 0.25% L-Arg + 100 mg/kg Vit E); and Diet 6: (PO and SO blend + 0.25% L-Arg + 150 mg/kg Vit E)*.

**Table 2:** Feed composition and calculated nutrient contents of the experimental diets fed to broiler chickens during the finisher period.

**Ingredient (g/kg)**	Dietary treatments^*^					
	**Diet 1 (control)**	**Diet 2 (positive control)**	**Diet 3**	**Diet 4**	**Diet 5**	**Diet 6**
Corn	495.8	490.9	490.9	490.9	490.9	490.9
Soybean meal	291.6	287.4	287.4	287.4	287.4	287.4
Palm oil (PO)	60.00	40.0	40.0	40.0	40.0	40.0
Sunflower oil (SO)	0.00	20.0	20.0	20.0	20.0	20.0
Wheat bran	34.0	45.5	45.5	45.5	45.5	45.5
Wheat pollard	50.5	43.4	43.4	43.4	43.4	43.4
Fish meal	27.7	30.4	30.4	30.4	30.4	30.4
L-Lysine	2.00	2.00	2.00	2.00	2.00	2.00
DL-Methionine	2.00	2.00	2.00	2.00	2.00	2.00
DCP^1^	18.2	18.3	18.3	18.3	18.3	18.3
Calcium carbonate	9.80	9.30	9.30	9.30	9.30	9.30
Choline chloride	0.800	0.800	0.800	0.800	0.800	0.800
Salt	3.00	3.00	3.00	3.00	3.00	3.00
Mineral premix^2^	1.50	1.50	1.50	1.50	1.50	1.50
Vitamin premix^3^	1.50	1.50	1.50	1.50	1.50	1.50
Antioxidant^4^	0.200	0.200	0.200	0.200	0.200	0.200
Toxin binder^5^	1.30	1.30	1.30	1.30	1.30	1.30
Arginine	0.000	2.50	2.50	2.50	2.50	2.50
Vit E (mg/kg)	–	–	20.0	50.0	100	150
**Chemical composition (Calculated)^6^**						
ME (MJ/kg)	13.0	13.0	13.0	13.0	13.0	13.0
CP (g/kg)	195	195	195	195	195	195
Fat (g/kg)	83.1	84.0	84.0	84.0	84.0	84.0
Fiber (g/kg)	40.4	40.5	40.5	40.5	40.5	40.5
Calcium (g/kg)	12.2	12.2	12.2	12.2	12.2	12.2
Available (g/kg)	4.60	4.60	4.60	4.60	4.60	4.6
Lysine (g/kg)	12.3	12.3	12.3	12.3	12.3	12.3
Methionine (g/kg)	5.30	5.30	5.30	5.30	5.30	5.30
Arginine (g/kg)	12.8	15.3	15.3	15.3	15.3	15.3
Meth + cyst (g/kg)	8.30	8.30	8.30	8.30	8.30	8.30
Tryptophan (g/kg)	2.40	2.40	2.40	2.40	2.40	2.40
α-tocopherol (analyzed, mg/kg)	39.4	36.6	47.5	77.4	122	164

1 *Di-calcium phosphate*.

2 *Vitamin premix contained: retinol 2 mg, α-tocopherol 0.02 mg, cholecalciferol 0.03 mg, menadione 1.33 mg, thiamin 0.83 mg, cobalamin 0.03 mg, riboflavin 2.0 mg, biotin 0.03 mg, folic acid 0.33 mg, niacin 23.30 mg, pantothenic acid 3.75 mg, and pyridoxine 1.33 mg*.

3 *Mineral premix contained: Fe 120 mg, Zn 100 mg, Mn 150 mg, Cu 20 mg, Mg 12 mg, Co 0.6 mg, and Se 0.20 mg*.

4 *Antioxidant contained: butylatedhydroxyanisole (BHA)*.

5 *Toxin binder contained: natural hydrated sodium calcium aluminum silicates (HSCAS)*.

6 *The rations were formulated using FeedLIVE software (Thailand)*.

* *Diet 1: 6% palm oil (negative control); Diet 2: PO and SO blend (4% palm oil and 2% sunflower oil) + 0.25% L-Arg (positive control); Diet 3: (PO and SO blend + 0.25% L-Arg + 20 mg/kg Vit E); Diet 4: (PO and SO blend + 0.25% L-Arg+ 50 mg/kg Vit E); Diet 5: (PO and SO blend + 0.25% L-Arg + 100 mg/kg Vit E); and Diet 6: (PO and SO blend + 0.25% L-Arg + 150 mg/kg Vit E)*.

### Sample and Data Collection

The body weight and feed intake of the broilers were measured weekly to calculate body weight gain (BWG) and feed conversion ratio (FCR). At 21 days, blood samples were collected for measurement of IgG and IgM. At 42 days, 12 broilers per treatment were slaughtered for sampling of blood, breast muscle, abdominal fat, and internal organs. Breast meat samples were kept in a −20°C freezer for analysis of chemical composition and α-tocopherol content of the meat. Spleen samples were snapped in liquid nitrogen and stored in a −80°C freezer for the measurement of cytokine expression. Internal organs and abdominal fat were collected to estimate their percentage, while serum and plasma samples were collected for the measurement of immunoglobulin (IgG and IgM) and lipid profile, respectively.

### Determination of Vit E

The Vit E (α-tocopherol) content of meat samples was determined according to the method of Rutkowski and Grzegorczyk (24). 0.5 g of pulverized sample was placed into a screw cap centrifuged tube to which was added 0.5 mL of anhydrous ethanol followed by a vigorous mixing for 1 min. Approximately 3 mL of xylene was added into the tube and mixed thoroughly for 1 min. The tube was subsequently centrifuged at 1,500 × g for 10 min. After centrifugation, 1.5 mL of the upper layer (extract) was transferred to a separate test tube and 0.25 mL of a 6.02 mM solution of batophenanthroline was added and mixed in. Subsequently, test and standard samples were prepared, and absorbance was measured against a blank at 539 nm using a spectrophotometer (Secomam, Domont, France). The α-tocopherol content of meat samples was measured using the following formula as the amount of α-tocopherol: (μM) = (Ax/As) × Cs, where, Cs = concentration of standard; Ax = Absorbance of test sample; and As = Absorbance of standard.

### Proximate Analysis of Meat

The breast meat samples were analyzed for moisture, ash, crude fat (EE), and crude protein content following the methods of AOAC (25).

### Plasma Lipid Profile, Blood Protein, Albumin, and Liver Enzyme Estimation

At 42 days of age, blood samples were collected from six chickens per treatment into separate vacutainer tubes containing ethylenediaminetetraacetic acid (EDTA). The blood samples were mixed by inverting the tubes gently and were then stored in ice before centrifugation. Plasma was separated by centrifuge at 3,000 × g for 10 min, then was transferred into vials and stored at −80°C for further analysis. The plasma lipid profiles, aspartate amino transferase (AST), alanine amino transferase (ALT), alkaline phosphatase (ALP), total protein, and albumin were measured using an Automatic Analyzer 902 (Hitachi, Germany). Serum very low density lipoprotein-cholesterol (VLDL-C) and LDL-C were determined according to the Friedewald Equation as described by Loh et al. (26).

### Immune Response

The IgG and IgM concentrations were estimated using Chicken IgM ELISA Kits (Immunology Consultants Laboratories, Inc., USA) and Chicken IgG ELISA Kits (CEA544Ga, Cloud-Clone Corp., USA), respectively. Serum samples were removed from the freezer and allowed to thaw and were then mixed gently by inverting. The samples were diluted based on an expected concentration of the analyte to fall within the ranges of the standard curve. The IgM standard was prepared by adding 1 mL de-ionized water into the supplied IgM calibrator in order to obtain a concentration of 54.20 μg/mL. The tubes were labeled for the preparation of IgM standard at 100, 50, 25, 12.5, 6.25, and 3.125 ng/mL using the supplied diluent. A duplicate 100 μL of sample, blank, and standard were placed into pre-assigned wells and incubated for 30 min and then washed four times with wash solutions. After that, 100 μL of diluent enzyme antibody conjugate was added to each well, covered, and incubated at 22°C for 30 min, and washed four times. TMB substrate (3, 3′, 5, 5′-tetramethyl benzidine) solution was dispensed into each well and kept in the dark at room temperature for 10 min. Finally, 100 μL of 0.3 M sulfuric acid (stop solution) was added into each well, and absorbance of the solution was measured at 450 nm using a microplate reader (Model 3550-UV, Bio-Rad). Serum IgM concentrations in each sample were estimated using standard curves which were obtained by plotting the OD450 against standard concentrations.

The IgG concentration of serum samples was determined by using Chicken IgG ELISA Kits. The serum samples were thawed and diluted 2,000-fold by using 0.01 mol/L PBS (pH = 7.0–7.2). The standard was prepared by diluting stock to obtain a concentration of stock solution 100 μL/mL and then diluted at 100, 33.33, 11.11, 3.70, and 1.23 ng/mL. Aliquots of 50 μL of respective standard, sample, and blank in duplicate were dispensed into the previously selected wells; subsequently, the same amount (μL) of diluent A was added and the solution was incubated at 37°C for 1 h. After 1 h, the solution was washed four times and diluent B (100 μL) was added, followed by an incubation period of 30 min at 37°C. The solution was then washed and 90 μL of substrate solution (TMB) was added; it was then covered and incubated in the dark at 37°C for 15 min. Thereafter, 50 μL of 0.3 M sulfuric acid (stop solution) was added into each well, and absorbance was measured at 450 nm using a microplate reader (Model 3550-UV, Bio-Rad). Serum IgG concentrations were calculated using the equation from the standard curves. The concentrations of IgM and IgG were expressed as ng/mL of blood serum.

### RNA Isolation and Real Time PCR

Spleen samples were collected immediately after slaughter, snap frozen in liquid nitrogen, and stored in a −80°C freezer until used for RNA isolation. About 30 mg of splenic frozen tissue from each bird was homogenized and total RNA was extracted and purified using an RNeasy plus Mini kit (QIAGEN, Germany) according to the manufacturer's protocol. The purity and concentration of RNA was assessed using a NanoDrop ND-1000 UV-Vis Spectrophotometer at absorbance 260/280 nm, and absorbance ranged from 1.9 to 2.1 for all samples. Total RNA was then reverse transcribed to cDNA using a QuantiNova™ Reverse transcription kit (Qiagen, Hilden, Germany) as described in the manufacturer's protocol. Then, 1 μg of purified RNA was reacted with 4 μL of RT buffer, 1 μL transcriptase, 1 μL RT primer mix, 2 μL gDNA wipeout buffer, and RNase free water to produce a final volume of 20 μL.

Cytokine expression was performed with real time PCR (Bio-Rad, Hercules, CA. USA) using a QuantinovaTM SYBRR Green PCR kit (Qiagen) according to the manufacturer's instructions. The mRNA of β-actin (QT00600614, Qiagen) was used as the internal control gene to normalize the target genes. Real time qPCR analysis was carried out using a QuantiTech Primer Assay (200) [IFN (Cat. QT00598059), TGF-ß1 (Cat.QT00644049), TNF-α (Cat.QT00596106), and IL8 (Cat. QT00600446), Qiagen]. Each reaction (20 μL) comprised of 10 μL SYBR Green PCR Master mix, 1 μL cDNA, 1 μL of each (forward and reverse) primer, and 7 μL of RNase free water. Thermal cycling was initiated at 95°C for 2 min. Subsequently, 40 PCR cycles of denaturation for 5 s at 94°C were run, followed by annealing and extension for 10 s at 59°C. Each sample was run in duplicate and the mean of the duplicates was used to allocate cycle threshold (Ct) values. The concentration of test genes in relation to those of the reference gene (β-actin) mRNA expression were estimated based on comparative Ct value methods. The 2^−ΔΔC(t)^ method was used to determine the relative changes in the mRNA expression of genes estimated from real time PCR (27).

### Statistical Analysis

The experiments followed a completely randomized design (CRD). All data for broiler performance, carcass characteristics, IgG and IgM concentration, and gene expression were statistically analyzed using PROC GLM of SAS (28) using the following model:

$$Y_{\text{ij}}=\mu +F_{\text{i}}+e_{\text{ij}}$$

Where, Y_ij_ is the dependent variable; μ is overall mean; F_i_ is the effect of dietary treatment (i = 1 to 6); and e_ij_ random error associated with each record, distributed as N (0, σ^2^).

The replicate was the experimental unit. The means were separated using Tukey's Honestly Significant Difference test and the level of significance was set at *P* < 0.05.

## Results

### Broiler Performance

The performance of broilers fed a dietary oil blend, supplemented with 0.25% L-Arg, and different levels of Vit E is shown in Table 3. Body weight and body weight gain were higher for broilers fed the PO and SO blend and supplemented with L-Arg and different levels of Vit E than for those fed the control diet (Diet 1) during the finisher period whereas in the starter period Diet 4 and 6 showed significantly higher values of these parameters. Moreover, FCR was greater (*P* < 0.05) in broilers fed the control diet than those fed other dietary treatments. However, no significant differences (*P* > 0.05) were found in body weight gain and FCR between the broilers fed different levels of Vit E compared to those fed the positive control diet (Diet 2).

**Table 3:** Growth performance of broilers fed PO and SO blend, supplemented with 0.25% L-Arg and different levels of Vit E.

**Parameter**	Treatments^*^						**SEM**
	**Diet 1 (control)**	**Diet 2 (positive control)**	**Diet 3**	**Diet 4**	**Diet 5**	**Diet 6**	
**1–21 days**							
Initial body weight (g)	41.00	41.36	41.61	40.14	40.39	40.33	0.034
Body weight (g)	669^b^	745^a^	723^a^	744^a^	715^ab^	738^a^	1.14
Body weight gain (g)	628^b^	704^a^	681^ab^	704^a^	675^ab^	698^a^	6.85
Feed intake (g)	1072	1082	1061	1081	1070	1099	8.46
FCR	1.71^a^	1.54^b^	1.56^b^	1.54^b^	1.59^b^	1.58^b^	0.015
**22–42 days**							
Body weight (g)	2316^b^	2577^a^	2542^a^	2580^a^	2551^a^	2530^a^	3.53
Body weight gain (g)	1647^b^	1832^a^	1819^a^	1836^a^	1835 ^a^	1792^a^	14.8
Feed intake (g)	2834	2913	2894	2851	2933	2905	13.6
FCR	1.72^a^	1.59^b^	1.59^b^	1.55^b^	1.60^b^	1.62^b^	0.011

a,b,c *Means with different superscripts in the same row differ significantly (P < 0.05)*.

* *Diet 1: 6% palm oil (negative control); Diet 2: PO and SO blend (4% palm oil and 2% sunflower oil) + 0.25% L-Arg (positive control); Diet 3: (PO and SO blend + 0.25% L-Arg + 20 mg/kg Vit E); Diet 4: (PO and SO blend + 0.25% L-Arg+ 50 mg/kg Vit E); Diet 5: (PO and SO blend + 0.25% L-Arg + 100 mg/kg Vit E); and Diet 6: (PO and SO blend + 0.25% L-Arg + 150 mg/kg Vit E)*.

*SEM, standard error of the means*.

### Carcass Characteristics and Internal and Lymphoid Organs

The effects of a dietary PO and SO blend + 0.25% L-Arg + different levels of Vit E on carcass characteristics and internal and lymphoid organs of broiler chickens are presented in Table 4. The broilers fed Diet 1 had significantly (*P* < 0.05) lower carcass and breast meat yields compared to those fed other diets. However, no significant differences were observed between chickens supplemented with different levels of Vit E (Diets 3–6) and Diet 2. The leg yield and the internal organs (liver, gizzard, and heart) percentage did not differ (*P* > 0.05) among the dietary treatments. However, the abdominal fat percentage was higher (*P* < 0.05) in chickens fed the control diet compared to other dietary treatments. The lymphoid organs (spleen and bursa of Fabricius weight) were not influenced (*P* > 0.05) by the dietary treatments.

**Table 4:** Carcass characteristics and internal and lymphoid organs of broilers fed PO and SO blend end, supplemented with 0.25% L-Arg, and different levels of Vit E.

**Parameter**	Treatments^*^						**SEM**
	**Diet 1**	**Diet 2**	**Diet 3**	**Diet 4**	**Diet 5**	**Diet 6**	
Carcass yield (%)	74.1^b^	77.6^a^	78.7^a^	79.0^a^	78.5^a^	78.6^a^	0.337
Breast yield (%)	32.2^b^	34.8^ab^	35.2^ab^	36.8^a^	35.3^a^	36.7^a^	0.398
Leg yield (%)	25.3	26.1	26.5	26.1	27.0	26.2	0.230
Abdominal fat (%)	1.76^b^	1.57^a^	1.53^a^	1.52^a^	1.52^a^	1.56 ^a^	0.022
Gizzard (%)	2.25	2.45	2.23	2.24	2.44	2.39	0.049
Heart (%)	0.390	0.390	0.430	0.420	0.410	0.420	0.087
Bursa of Fabricius (%)	0.090	0.100	0.110	0.120	0.120	0.110	0.003
Spleen (%)	0.100	0.110	0.130	0.130	0.140	0.120	0.003

a,b,c *Means with different superscripts in the same row differ significantly (P < 0.05)*.

* *Diet 1: 6% palm oil (negative control); Diet 2: PO and SO blend (4% palm oil and 2% sunflower oil) + 0.25% L-Arg (positive control); Diet 3: (PO and SO blend + 0.25% L-Arg + 20 mg/kg Vit E); Diet 4: (PO and SO blend + 0.25% L-Arg+ 50 mg/kg Vit E); Diet 5: (PO and SO blend + 0.25% L-Arg + 100 mg/kg Vit E); and Diet 6: (PO and SO blend + 0.25% L-Arg + 150 mg/kg Vit E)*.

*SEM, standard error of the means*.

### Vit E (α-Tocopherol) Concentration of Breast Meat

The Vit E concentration of breast meat taken from broilers fed a dietary PO and SO blend, supplemented with 0.25% L-Arg, and different levels of Vit E, is shown in Table 5. The α-tocopherol concentration of meat was significantly increased along with increased levels of α-tocopherol in the diet. The concentration of α-tocopherol in meat increased noticeably when the broiler diet was supplemented with at least 50 mg/kg or more of Vit E.

**Table 5:** Vitamin E (α-tocopherol; mg/kg) concentration of the meat of broilers fed PO and SO blend, supplemented with 0.25% L-Arg, and different levels of Vit E.

Dietary Treatments^*^	**Vitamin E (mg/kg)**
Diet 1	5.67^e^ ± 0.087
Diet 2	5.14^f^ ± 0.075
Diet 3	6.37^d^ ± 0.101
Diet 4	10.1^c^ ± 0.110
Diet 5	13.1^b^ ± 0.091
Diet 6	13.7^a^ ± 0.106

a,b,c *Means with different superscripts in the same column differ significantly (P < 0.05)*.

* *Diet 1: 6% palm oil (negative control); Diet 2: PO and SO blend (4% palm oil and 2% sunflower oil) + 0.25% L-Arg (positive control); Diet 3: (PO and SO blend + 0.25% L-Arg + 20 mg/kg Vit E); Diet 4: (PO and SO blend + 0.25% L-Arg+ 50 mg/kg Vit E); Diet 5: (PO and SO blend + 0.25% L-Arg + 100 mg/kg Vit E); and Diet 6: (PO and SO blend + 0.25% L-Arg + 150 mg/kg Vit E)*.

*The values are all presented as mean ± standard error*.

### Chemical Composition of Breast Meat

The chemical composition of breast muscle in broilers fed a dietary PO and SO blend, supplemented with 0.25% L-Arg and different levels of Vit E, is presented in Table 6. The percentage of moisture, ash, and fat content of breast meat were unaffected across the dietary treatments. The crude protein content of the breast meat of broilers fed Diet 1 was lower (*P* < 0.05) than those of broilers fed other dietary treatments.

**Table 6:** Chemical composition of the breast meat of broilers fed PO and SO blend, supplemented with 0.25% L-Arg, and different levels of Vit E.

**Parameter (%)**	Treatments^*^						**SEM**
	**Diet 1**	**Diet 2**	**Diet 3**	**Diet 4**	**Diet 5**	**Diet 6**	
Moisture	70.6	69.0	69.2	68.9	68.7	68.8	0.252
Ash	1.59	1.63	1.62	1.63	1.62	1.63	0.005
Crude protein	23.7^b^	25.9^a^	25.9^a^	26.1^a^	26.4^a^	26.2^a^	0.276
Crude fat	3.60	3.24	3.14	3.11	3.05	3.13	0.090

a,b,c *Means with different superscripts in the same row differ significantly (P < 0.05)*.

* *Diet 1: 6% palm oil (negative control); Diet 2: PO and SO blend (4% palm oil and 2% sunflower oil) + 0.25% L-Arg (positive control); Diet 3: (PO and SO blend + 0.25% L-Arg + 20 mg/kg Vit E); Diet 4: (PO and SO blend + 0.25% L-Arg+ 50 mg/kg Vit E); Diet 5: (PO and SO blend + 0.25% L-Arg + 100 mg/kg Vit E); and Diet 6: (PO and SO blend + 0.25% L-Arg + 150 mg/kg Vit E)*.

*SEM, standard error of the means*.

### Total Protein and Albumin Contents in Blood

**Figure 1:**
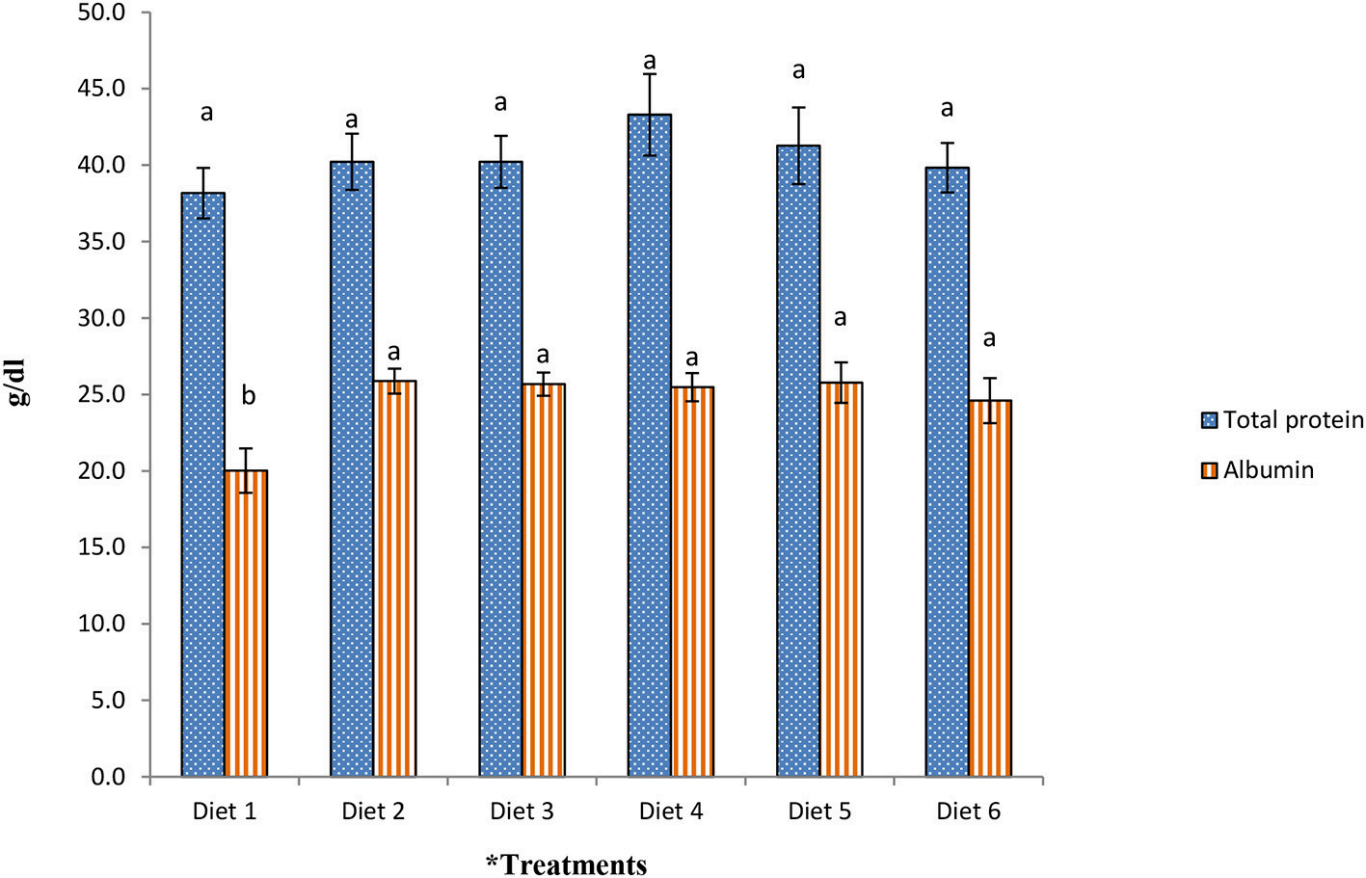
Effect of the supplementation of PO and SO blend, 0.25% L-Arg, and different levels of Vit E on plasma total protein and albumin content of broilers. ^a,b,c^Bars with the same color and no common letter differ significantly (*P* < 0.05). ^*^Diet 1: 6% palm oil (negative control); Diet 2: PO and SO blend (4% palm oil and 2% sunflower oil) + 0.25% L-Arg (positive control); Diet 3: (PO and SO blend + 0.25% L-Arg + 20 mg/kg Vit E); Diet 4: (PO and SO blend + 0.25% L-Arg+ 50 mg/kg Vit E); Diet 5: (PO and SO blend + 0.25% L-Arg + 100 mg/kg Vit E); and Diet 6: (PO and SO blend + 0.25% L-Arg + 150 mg/kg Vit E).

The total protein and albumin contents in blood plasma in broilers fed a dietary PO and SO blend, supplemented with 0.25% L-Arg and different levels of Vit E, are illustrated in Figure 1. No significant difference (*P* > 0.05) was observed in total plasma protein among the dietary treatments, although numerically higher total protein levels were found in the plasma of broilers fed a diet supplemented with either 0.25% L-Arg alone or in combination with Vit E. The chickens fed Diet 1 had a lower (*P* < 0.05) plasma albumin level than those fed other diets.

### IgG and IgM Concentrations in Blood Serum

The effects of supplementation of a dietary PO and SO blend, supplemented with 0.25% L-Arg and different levels of Vit E, on serum IgG and IgM concentrations for 21 days old broilers are presented in Table 7. Serum IgG and IgM concentrations increased (*P* < 0.05) for broilers fed the above mentioned supplements compared to the control. Moreover, chickens fed an oil-blend diet with 0.25% L-Arg combination and Vit E amounts ranging from 50mg to 150 mg/kg (Diet 3–6) had higher (*P* < 0.05) IgG and IgM concentrations than other dietary treatments; however, IgG and IgM concentrations were statistically similar (*P* > 0.05) among broilers fed Diets 3–6.

**Table 7:** Effect of the supplementation of PO and SO blend, L-Arg, and different levels of Vit E on serum IgM and IgG (log_10_) concentrations in broilers at 21 days of age.

**Parameter**	Treatments^*^						**SEM**
	**Diet 1**	**Diet 2**	**Diet 3**	**Diet 4**	**Diet 5**	**Diet 6**	
IgG	2.98^b^	3.01^ab^	3.01^ab^	3.03^a^	3.04^a^	3.04^a^	0.006
IgM	2.71^b^	2.82^ab^	2.89^a^	2.90^a^	2.90^a^	2.89^a^	0.017

a,b,c *Means with different superscripts in the same row differ significantly (P < 0.05)*.

* *Diet 1: 6% palm oil (negative control); Diet 2: PO and SO blend (4% palm oil and 2% sunflower oil) + 0.25% L-Arg (positive control); Diet 3: (PO and SO blend + 0.25% L-Arg + 20 mg/kg Vit E); Diet 4: (PO and SO blend + 0.25% L-Arg+ 50 mg/kg Vit E); Diet 5: (PO and SO blend + 0.25% L-Arg + 100 mg/kg Vit E); and Diet 6: (PO and SO blend + 0.25% L-Arg + 150 mg/kg Vit E)*.

*SEM, standard error of the means*.

### Blood Lipid Profile and Liver Enzyme Function

The effects of a dietary PO and SO blend, supplemented with 0.25% L-Arg and different levels of Vit E, on blood lipid profiles and liver enzymes in broilers are shown in Table 8. The concentrations of total cholesterol, TG, and VLDL-C (mmol/L) were greater (*P* < 0.05) in the blood plasma of broilers fed Diet 1 compared with those fed other dietary treatments, whereas the LDL-C (mmol/L) was significantly lower in Diet 6 than other dietary treatments. The level of HDL-C was not affected (*P* > 0.05) by dietary treatment, although the highest level (2.28 mmol/L) was found in broilers fed Diet 2 and Diet 5. In contrast, the lowest level (2.13 mmol/L) was observed in Diet 1. Dietary treatments did not affect (*P* > 0.05) alanine amino transferase (ALT) and aspartate amino transferase (AST) in broilers.

**Table 8:** Effect of dietary supplementation of PO and SO blend, L-Arg, and different levels of Vit E on plasma lipid profiles and liver function of broilers at 42 days of age.

**Parameter**	Treatments^*^						**SEM**
	**Diet 1**	**Diet 2**	**Diet 3**	**Diet 4**	**Diet 5**	**Diet 6**	
**Lipid profile (mmol/L)**							
CHOL	3.79^a^	3.31^b^	3.31^b^	3.27^b^	3.26^b^	3.02^b^	0.065
TG	0.780^a^	0.540^ab^	0.550^ab^	0.520^b^	0.500^b^	0.500^b^	0.029
HDL-C	2.13	2.28	2.24	2.20	2.28	2.25	0.051
VLDL-C	0.150^a^	0.100^b^	0.110^b^	0.110^b^	0.100^b^	0.100^b^	0.006
LDL-C	1.51^a^	0.930^ab^	0.960^ab^	0.960^ab^	0.880^ab^	0.670^b^	0.076
**Liver function**							
Alt (U/L)	5.05	4.72	4.83	4.80	4.45	4.08	0.219
Ast (U/L)	329	284	287	320	290	310	8.76

a,b,c *Means with different superscripts in the same row differ significantly (P < 0.05)*.

* *Diet 1: 6% palm oil (control), Diet 2: (6% PO and SO blend + 0.25% L-Arg; positive control), Diet 3: (6% PO and SO blend + 0.25% L-Arg + 20 mg/kg Vit E), Diet 4: (6% PO and SO blend + 0.25% L-Arg + 50 mg/kg Vit E), Diet 5: (6% PO and SO blend + 0.25% L-Arg + 100 mg/kg Vit E), Diet 6: (6% PO and SO blend + 0.25% L-Arg + 150 mg/kg Vit E)*.

*SEM, standard error of the means*.

### Splenic mRNA Expression

**Figure 2:**
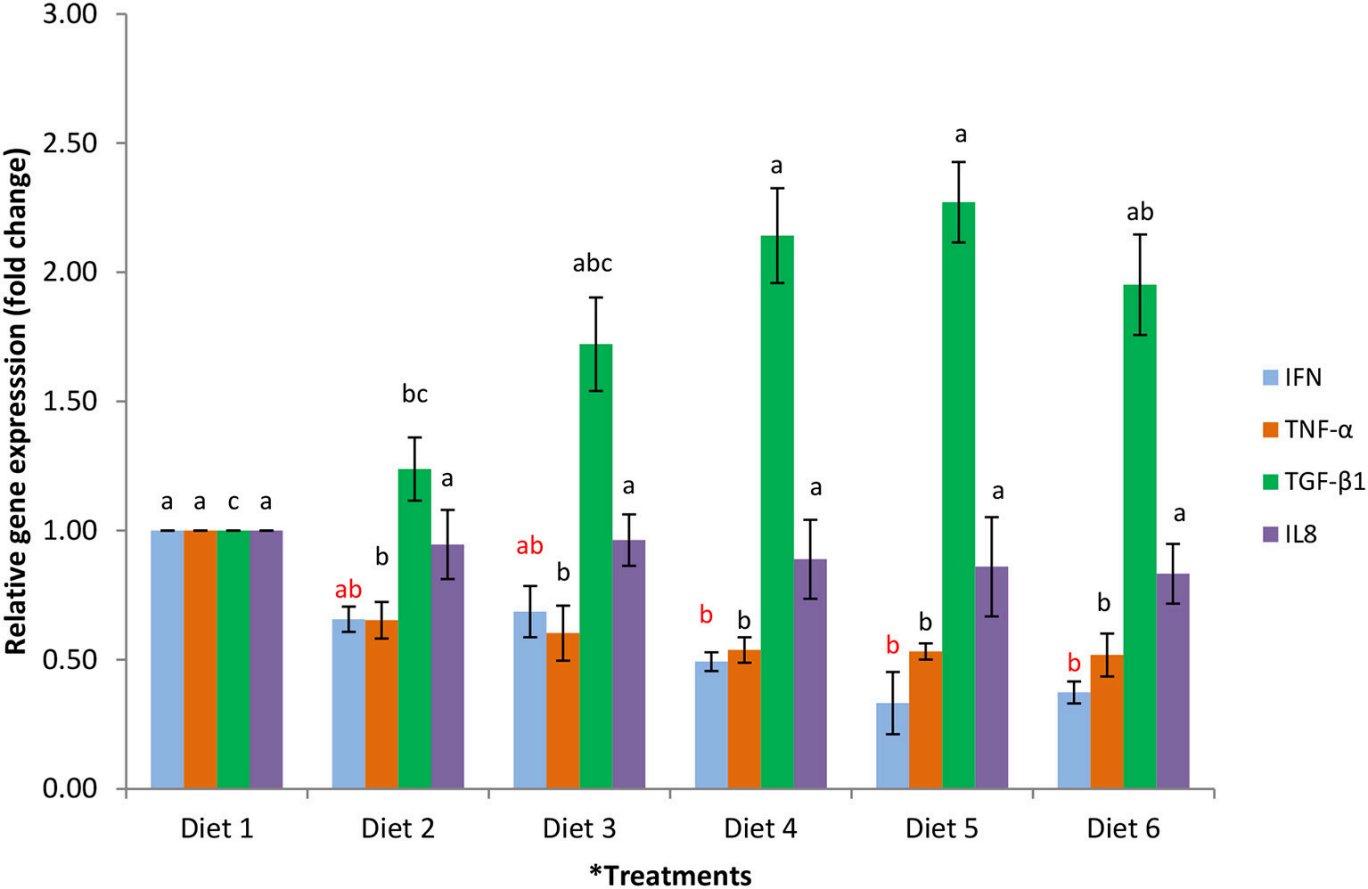
Comparison of IFN, TNF-α, TGF-β1, and IL8 expression in the spleen tissue of broiler chickens fed PO and SO blend, supplemented with 0.25% L-Arg and different levels of Vit E. Values were normalized with a housekeeping gene, β-actin. Subsequently, treated samples were expressed relative to gene expression of the control group (Diet 1). ^*^Diet 1: 6% palm oil (negative control); Diet 2: PO and SO blend (4% palm oil and 2% sunflower oil) + 0.25% L-Arg (positive control); Diet 3: (PO and SO blend + 0.25% L-Arg + 20 mg/kg Vit E); Diet 4: (PO and SO blend + 0.25% L-Arg+ 50 mg/kg Vit E); Diet 5: (PO and SO blend + 0.25% L-Arg + 100 mg/kg Vit E); and Diet 6: (PO and SO blend + 0.25% L-Arg + 150 mg/kg Vit E). ^a,b,c^Bars with the same color and no common letter differ significantly (*P* < 0.05).

Splenic mRNA expression in broilers fed a dietary PO and SO blend, supplemented with 0.25% L-Arg and different levels of Vit E, is shown in Figure 2. Dietary treatments did not affect (*P* > 0.05) the expression of IL8 in broilers. The expression of IFN in those broilers fed Diet 1 was greater (*P* < 0.05) when compared with those fed other dietary treatments. The expression of IFN decreased (*P* < 0.05) as the level of Vit E increased in the diet. Diet 1 upregulated the expression of TNF-α in broilers compared with other dietary treatments. Diet 1 down-regulated the expression of TGF-ß1 in broilers compared to those fed Diets 4, 5, and 6. The expression of TGF-ß1 in the broilers fed Diets 2 and 3 did not differ from those fed other dietary treatments. Moreover, no differences were observed in TGF-ß1, IFN, and TNF-α mRNA expression among broilers fed the PO and SO blend, supplemented with 0.25% L-Arg and Vit E ranging from 50 to 150 mg/kg feed (Diets 4–6).

## Discussion

### Performance of Chickens

Broilers fed a diet containing an oil blend of SO and PO, 0.25% L-Arg, and different levels of Vit E had a higher body weight, greater body weight gain, and a lower FCR compared to those fed the control diet (Diet 1). These results might be due to the higher levels of unsaturated fats in the oil blend compared to only PO present in Diet 1. Improved growth rates and FCR have been reported for broilers fed diets containing higher proportions of long-chain fatty acids, due to their high energy-yielding capacity and digestibility compared to diets rich in saturated fats (4). In addition, supplementation of 0.25% L-Arg in the diet also plays an important role in the growth of broilers as L-Arg is involved in the biosynthesis of protein, glutamine, polyamines, ornithine, and proline. Wu et al. (9) state that L-Arg augments body weight gain in ducks. Furthermore, dietary inclusion of an oil blend (castor oil) is reported as a good option for increasing growth performance, even in coccidiosis-challenged broilers (29). In this aspect, the current findings suggest a synergistic effect among oil blend, L-Arg, and Vit E in increasing body weight gain and decreasing FCR during days 1–21 and 22–42 of age. Likewise, growth performance and FCR improved linearly when broilers were fed a diet containing a combination of canola oil (rich in PUFA) and 2–4 g/kg L-Arg (11).

### Carcass Characteristics, Internal, and Lymphoid Organs

The carcass and breast muscle yield of broilers was significantly increased and abdominal fat was decreased in diets supplemented with PO and SO blend and 0.25% L-Arg, compared to the control diet. The carcass yield stimulation effect could be credited to L-Arg as it is involved in the synthesis of protein, proline, and hydroxyproline, which are essential for the formation of connective tissue, particularly through the gain of breast muscle and reduction of abdominal fat via alterations in carbohydrate and fat metabolism. Jiao et al. (30) report that supplementation of dietary Arg could enhance muscle growth and increase carcass and breast yield in broilers. Due to its faster accumulation, abdominal fat content is used as an indicator to assess total body fat content in broiler chickens (31). The dietary inclusion of oils containing long chain fatty acid, such as SO, soybean oil, and combinations of PO and SO (oil blend), enhanced oxidation and reduced the synthesis of fatty acids, resulting in less accumulation of abdominal fat in broilers (1, 4). The significant reduction of abdominal fat and higher carcass yield reported in the present study could also be due to the combined effects of L-Arg and the PO and SO blend inhibiting the expression of hepatic FASmRNA and ACCmRNA, which suppresses the synthesis of fatty acids and leads to less abdominal fat deposition (12).

The size of the bursa of Fabricius was not affected by Vit E supplementation, and no significant differences were found between the dietary treatments. Similar results were observed by Rostami et al. (32) and El-Gogary (33) who report that the dietary supplementation of Vit E or rosemary does not affect the size of the bursa of Fabricius or the weight of other lymphoid tissues. However, Biswas et al. (34) report that the supplementation of combined L-Arg and Vit E in broiler diets changed the size of the bursa of Fabricius. The relative spleen weight in the current study was not influenced by dietary treatments. This observation corroborates with the findings of Abdulkalykova and Ruiz-Feria (35), who observed that the supplementation of L-Arg and Vit E had no effect on the weight of spleens in broilers.

### Tocopherol Content of Breast Muscle

The inclusion of antioxidants, particularly Vit E, in the diet is an important approach to increase the antioxidant capacity of meat to prevent lipid oxidation. In the present study, the α-tocopherol content of meat increased as the level of Vit E increased in the diet. Similarly, Skrivan et al. (36) report that dietary supplementation of higher levels of α-tocopherol increased the α- tocopherol content in both the thigh and breast muscle of broilers. In addition, Zhang et al. (37) reveal that increased levels of dietary α-tocopherol concentration significantly increased the α-tocopherol content of liver tissue and blood serum in chickens. The higher concentration of α-tocopherol in meat is helpful in preservation, as it protects sensitive compounds, such as UFA and PUFA, from oxidation. The increased Vit E concentration in the broiler diet increased Vit E content and decreased lipid peroxidation in meat, thus highlighting the benefits of additional Vit E for maintaining fat quality in broiler meat (38). Our recent findings report an effect of Vit E on lipid oxidation in broiler meat during the postmortem storage, and a significant reduction of MDA production in meat observed by adding 50–150 kg/mg Vit E with the oil blend of PO and SO and L-Arg (22).

### Chemical Composition of Breast Muscle

The proximate composition of meat can be altered through dietary manipulation. The present findings indicated that different levels of Vit E with PO and SO blend and 0.25% L-Arg did not influence the moisture, ash, and fat content of meat. However, supplementation of L-Arg with the PO and SO blend increased the crude protein content of breast meat. This observation could be because Arg promotes muscle development as opposed to fat deposition in carcasses. This observation concurs with that of Tan et al. (39), who report that Arg supplementation increases protein content in the skeletal muscle of pigs.

In the current study, supplementation of different levels of Vit E did not affect the protein content nor other proximate components in boiler breast meat. This finding aligns with those of Souza et al. (40), who observed that dietary fat and Vit E had no effect on the proximate composition of broiler breast meat.

### Blood Lipid Profile and Liver Enzyme Function

The results of the current study showed that broilers fed PO and SO blend and 0.25% L-Arg, alone or in combination with different levels of Vit E, had significantly decreased total cholesterol, TG, VLDL-C, and LDL-C as compared to the control. This observation might be due to increased levels of PUFAs in the oil blend, or because L-Arg inhibits the activity of stearoyl-CoA desaturase expression in the liver (12), which leads to a reduced level of triglycerides and VLDL secretion from the liver into the blood in chickens. These results are in agreement with those of Fouad et al. (10) and Velasco et al. (41) who report that serum total cholesterol, triglycerides, and LDL-C decreased in chickens following dietary supplementation of L-Arg and increased levels of SO (rich in PUFAs) in the diet, respectively. L-Arg could reduce blood cholesterol concentration through regulation of the metabolism and biosynthesis of cholesterol through the NO production pathway. On the other hand, in the current study the lipid profile did not differ among birds fed different levels of Vit E. Similarly, Ahmed et al. (42) report that dietary supplementation of antioxidants did not influence blood plasma cholesterol, triglycerides, or VLDL. In contrast, Gumus and Imik (43) observe that administration of Vit E reduces total cholesterol, triglycerides, HDL, and VLDL in heat stressed birds.

Concentrations of the liver enzymes ALT and AST are important indicators in the assessment of liver function and damage to hepatic cells. These enzymes also indicate abnormalities of the liver, which could develop due to various changes in physiological and medical conditions. In the present results, plasma ALT and AST concentration showed a trend toward decreased values in broilers fed diets containing the PO and SO blend, L-Arg, and Vit E compared to the control diet; however, no significant differences were observed among the treatments. This observation is supported by the findings of Ahmed et al. (42) who note that ALT and AST did not differ significantly among broilers supplemented with Vit E or synthetic antioxidants compared with a control diet. However, Attia et al. (44) report a favorable effect of PUFA on hepatic cell integrity and observed a decreased level of plasma AST when broilers were fed increased levels of PUFA (fish oil and a mixture of three oils). These differences among the studies might be due to differences in oil sources, Vit E levels, and environment and management practices of the birds.

### IgG and IgM Concentrations in Blood Serum

IgG and IgM concentrations were examined to study the immune status of broilers fed the PO and SO blend + 0.25% L-Arg + Vit E. Nutritional supplementation is an effective strategy to increase immune response and improve avian health (45). In the present study, it was observed that serum IgG and IgM concentrations increased in broilers fed the PO and SO blend + 0.25% L-Arg + Vit E. The higher level of immunoglobulins could be attributed to increased levels of PUFAs, 0.25% L-Arg, and Vit E in the broiler diets, as both L-Arg and Vit E influence the immune response in chickens. Vit E is an antioxidant which decreases the formation of free-radicals and shields free radical attacks on double bonds of UFA in lipids. The ability of Vit E to reduce oxidative stress might be through the prevention of oxidation of UFAs and also through the alteration of eicosanoid metabolism and modulation of transcription factors which enhance avian immunity. Muir et al. (46) report that Vit E increases IgA concentration in intestinal scraps against anti-T toxoid. Vit E is particularly essential for the inhibition of fatty acid oxidation. Fatty acids can act as immunoregulatory particles that facilitate membrane fluidity, cellular communication, and second messenger elaboration; furthermore, the inhibitory action of Vit E on the stability of fatty acids may itself be immunoregulatory (38).

In addition, L-Arg modulates immune responses as Arg is involved in the synthesis of polyamine and proline, which are responsible for the proliferation of lymphocytes and the repair of damaged tissues, respectively. Moreover, via the NO production pathway, Arg repairs tissues through the stimulation of vasodilatation and the modulation of immune response (47). Hence, in the present study, increased IgG and IgM concentrations might be due to the combined effect of L-Arg and Vit E on broiler immune response.

### Plasma Total Protein and Albumin Concentrations

Blood protein acts as a carrier to transport different substances, such as lipids, proteins, hormones, minerals, and vitamins, and also plays an important role in maintaining the osmotic pressure of blood. In the present study, supplementation of 0.25% L-Arg with the PO and SO blend increased plasma albumin levels compared to the control diet. This observation agrees with the findings of Emadi et al. (48) who report that the supplementation of L-Arg had no effect on total protein but significantly increased the albumin concentration in blood plasma. Attia et al. (49) report that heat stress negatively affected chicken immune status by decreasing the blood plasma protein, γ-globulin (innate immunity), and blood plasma albumin (non-specific immune protein), which was recovered by supplementation with Vit E alone or with a combination of Vit C. In the present study, greater levels of plasma albumin in broilers fed the combination of PO and SO blend + 0.25% L-Arg + Vit E might be due to the greater body weight of broiler chickens in these groups, which had a higher concentration of plasma protein compared to lighter broilers in the control group; this suggests an increased demand for maintaining lean tissue growth. A synergistic effect of PO and SO blend, L-Arg, and Vit E was observed in blood plasma albumin (non-specific immune-protein), meat crude protein content, serum IgG and IgM level, and Vit E concentrations of meat, which could explain the superior performance of broilers in these treatment groups.

### Cytokine Expression

A decrease in the incidence of disease reflects changes in immune regulation via cytokine secretion. Nutrients such as amino acids, vitamins, and minerals act as immunomodulators and can be used to alter the immune response to pathogens by various pathways. Among amino acids, Arg acts as an immunologic modulator in a non-inflammatory manner because of its function as a substrate in the immune system. Moreover, vitamins, particularly Vit E, are antioxidants and have been reported to influence immune function in birds, including humoral and cellular response (19, 50).

In the current study, feeding broilers a dietary combination of 0.25% L-Arg and 50–150 mg/kg Vit E decreased pro-inflammatory cytokines IFN and TNF-α. This observation could be due to the modulatory effect of L-Arg and Vit E, which enhance non-specific immunity in the body by non-specifically killing fungi, bacteria, tumor cells, and parasites, thereby decreasing the pathogenic load. Arg produces NO as a metabolic product, playing a key role in modulating the immune response and inflammation via various pathways (51). The reduction in the expression of IFN and TNF-α in L-Arg- and Vit E-fed broilers corroborates the findings of Wu et al. (52), who observed that dietary supplementation of Arg and glutamine decreased TNF and IL2 in growing pigs.

Moreover, cytokine TGF-ß plays a key role in T-lymphocyte development and has anti-inflammatory action. In the present study, the expression of TGF-ß1 increased in broilers fed the PO and SO blend supplemented with 0.25% L-Arg and 50–150 mg/kg Vit E. This result concurs with that of Kaiser et al. (20) who observe that supplementation of higher levels of natural Vit E improves anti-inflammatory TGF-ß expression in broiler chickens. Liu et al. (53) observe that chickens fed a combination of Arg and Vit E had higher heterophil, oxidative burst, monocyte, and lymphocyte proliferation as compared with birds fed control diets. It is reported that dietary supplementation of both natural and synthetic Vit E increases one of the most important immune and inflammatory mediators, cytokine IL-6, compared to cyclophosphamide immunosuppressed broilers; this indicates that Vit E could enhance the secretion of some cytokines and thus improve immune status (50). Hence, in the current study the combined effects of 0.25% L-Arg and Vit E might alter the expression of cytokines and influence immune response in broilers. However, more studies are required to fully understand the combined effects of PO and SO blend, Arg, and Vit E and their interactions under stress conditions and specific disease challenges (parasitic and virus) in broiler chickens.

## Conclusions

A broiler diet including an oil blend of PO and SO (4:2) supplemented with 0.25% L-Arg and 50 to 150 mg/kg Vit E improved performance, immunoglobulin level, and immune-related cytokine expression, while resulting in decreased fat deposition and VLDL-C content in the blood. This diet could be used to decrease fat deposition, enhance broiler performance, and improve immune function in broilers.

## Data Availability Statement

All datasets generated for this study are included in the article.

## Ethics Statement

All the procedure in this animal study was reviewed, followed and approved by the Institutional Animal Care and Use Committee, Universiti Putra Malaysia (UPM/IACUC/AUP-R081/2016).

## Author Contributions

JK and TL: conceptualization and investigation. JK, TL, HF, and HA: methodology. JK: data curation and drafting. JK, TL, and KK: formal analysis. TL and HA: supervision. JK, TL, HF, HA, and KK: review and editing. All authors have read and agreed to the published version of the manuscript.

## Conflict of Interest

The authors declare that the research was conducted in the absence of any commercial or financial relationships that could be construed as a potential conflict of interest.
